# Bloc auriculo-ventriculaire post chirurgie cardiaque: à propos de 23 cas

**DOI:** 10.11604/pamj.2014.19.297.4614

**Published:** 2014-11-17

**Authors:** Sahar Mouram, Ibtissam Fellat, Mohamed Cherti

**Affiliations:** 1 Service de Cardiologie B, Hopital d'Enfants, Faculté de Médecine et de Pharmacie de Rabat, Université Mohammed V- Souissi, Rabat, Maroc

**Keywords:** Bloc auriculo-ventriculaire, pacemaker, chirurgie cardiaque, Atrioventricular block, pacemaker, heart surgery

## Abstract

Le bloc auriculo-ventriculaire (BAV) représente une complication non négligeable de la chirurgie cardiaque. Il est responsable de séquelles lourdes et graves compromettant le pronostic de la maladie et conduisant à la mise en place d'un stimulateur cardiaque définitif. Il est primordial d’étudier et de déterminer les différents facteurs prédictifs de BAV post opératoire, son histoire naturelle, son incidence ainsi que le délai exact de la mise en place d'un pacemaker. Notre étude est une étude rétrospective descriptive à propos de 23 patients opérés pour chirurgie cardiaque sous circulation extracorporelle entre octobre 1989 et août 2010 ayant présentés des troubles conductifs auriculo-ventriculaires en post opératoire. Plusieurs facteurs de risque ont été étudiés dans notre série, liés surtout à l'atteinte directe du noeud auriculo-ventriculaire ou à l'ischémie myocardique. Le délai d'apparition du trouble conductif ainsi que le délai d'implantation on été également étudié. Plusieurs facteurs favorisants de survenue de BAV ont été identifiés liés essentiellement au type de la cardiopathie opérée avec une prédominance de la cardiopathie congénitale, d'autres facteurs ont été rapportés notamment la présence d'hypertrophie ventriculaire gauche (HVG) de troubles conductifs en préopératoire, une durée du clampage aortique et de CEC prolongée. La moitié des patients ont présenté un BAV immédiat. Le délai d'implantation par rapport à la date de la découverte du BAV varie dans notre série d'une implantation immédiate après le diagnostic positif (J0) à un délai d'implantation allant jusqu’à 57 jours. Plusieurs facteurs de risques déterminent la survenue de BAV post chirurgie cardiaque, leur connaissance est primordial ainsi que le délai exact de l'implantation du stimulateur cardiaque définitif.

## Introduction

La chirurgie cardiaque sous circulation extra corporelle conduit à un risque non négligeable de troubles conductifs auriculo-ventriculaires [1]. Ils représentent des séquelles lourdes et graves compromettant le pronostic de la maladie et conduisant parfois à l'implantation d'un stimulateur cardiaque définitif. Le mécanisme des troubles conductifs auriculo-ventriculaires post opératoire reste mal élucidé, témoignant des difficultés de prise en charge, et plus particulièrement du moment exact de l'implantation du pacemaker [2, 3] De ce fait, il est primordial d'identifier d'une part les facteurs prédictifs et d'autre part de comprendre leur mécanismes. L'incidence du BAV varie selon le type de chirurgie, elle est estimée entre 1 à 3% au cours des cardiopathies congénitales et varie entre 23 et 37% après chirurgie valvulaire [3, 4]. L'objectif principal de ce travail est de déterminer, les différents facteurs prédictifs de BAV de haut degré post opératoire, son histoire naturelle, son incidence ainsi que le délai exact pour mettre en place un pacemaker définitif.

## Méthodes

Il s'agit d'une étude rétrospective descriptive qui s’étend entre octobre 1989 et aout 2010 réalisée au service de la cardiologie B à propos de 23 patients. Le principal critère d'inclusion était: les patients opérés pour chirurgie cardiaque qu'elle soit congénitale, valvulaire ou pour pontage coronaire, ayant présenté en per opératoire en post opératoire immédiat ou tardif un bloc auriculo-ventriculaire de premier, deuxième ou troisième degré régressif ou persistant, ayant nécessité la mise en place ou non d'un stimulateur cardiaque définitif. Les différentes données per opératoires ont été recherchées systématiquement chez tous nos patients notamment: la durée de la circulation extra corporelle (CEC), du clampage aortique, la solution de la cardioplégie utilisée ainsi que le degré d'hypothermie. Le suivi évolutif était apprécié par la recherche de complications post opératoire notamment la survenue de BAV, son type, le mode d'installation, le délai du diagnostic d'implantation, l’évolution ainsi que les complications survenant après l'implantation du pacemaker.

## Résultats

**Figure 1:**
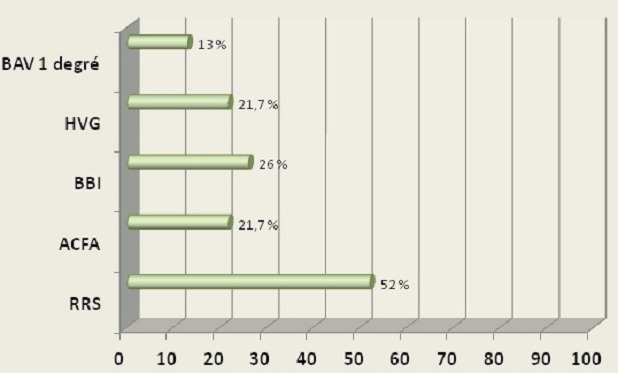
Répartition des malades selon les données de l'ECG initial

**Figure 2:**
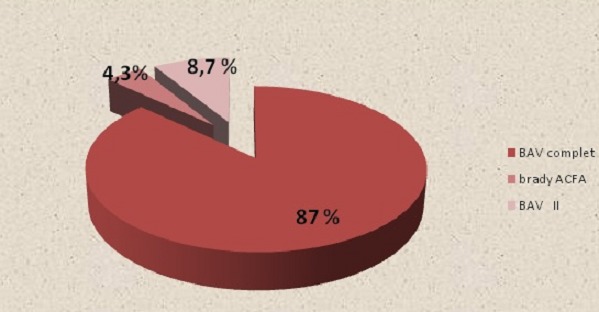
Répartition des malades selon le type du trouble conductif

La médiane d’âge de nos patients est de 33 ans avec des extrêmes allant de 4 à 80 ans. Le sexe ratio est de 15 hommes pour 8 femmes. La médiane du poids de nos malades est de 58,6 kg avec des extrêmes allant de 15 à 80 kg. 13 patients présentent des facteurs de risque cardiovasculaire. Une seule patiente âgée de 14ans est trisomique 21. Aucun patient n'a rapporté de syncope ou malaise lipothymique. 52% des malades sont initialement en rythme sinusal (Figure 1), 21% des patients sont en arythmie complète par fibrillation auriculaire 22% patients ont une hypertrophie ventriculaire gauche, 26% des patients ont un bloc de branche incomplet dont 2 patients avec un bloc de branche droit. 13% des patients avaient un BAV de 1 degré. Aucun patient n'a présenté d'hémi bloc ou de bloc tri fasciculaire en pré opératoire. La cardiopathie congénitale représente 52% de l'ensemble des indications suivie par les poly valvulopathies dans 26% des cas. 34.7% des patients ont eu un remplacement valvulaire aortique ( RVAo) 22% ont eu un remplacement valvulaire mitral (RVM) deux patients soit 8,6% ont eu une plastie mitrale alors que 22% ont une plastie tricuspidienne. La durée moyenne de la CEC est de 205,8 min concernant notre série. La durée du clampage aortique varie entre 70 min et 117 min pour une moyenne de 94,2 min. La solution de cardioplégie était à base de sang dans 72% des cas et de cristalloïdes dans 28% des cas. Des troubles conductifs de haut degré sont survenus chez 22 patients soit 95% des cas avec un cas de brady arythmie .87% des patients ont présenté un BAV complet, 8.7% des patients ont présenté un BAV 2ème degré type II (Figure 2).

**Figure 3:**
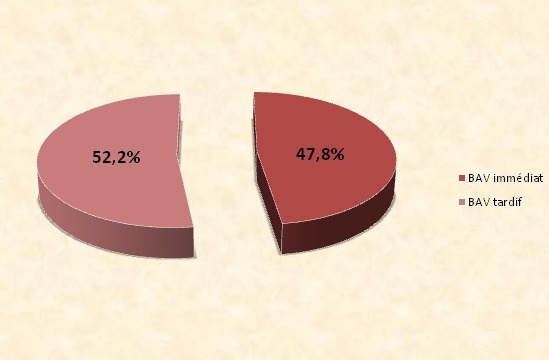
Répartition des malades selon le mode d'installation du trouble conductif

47.8% des patients ont présenté un BAV immédiat, dans les cas restant le BAV était tardif (Figure 3). Le délai du diagnostic a varié dans notre série entre: 0 jour et 3ans et 5 mois. Le délai d'apparition moyen est de 99 jours. Concernant la cardiopathie valvulaire, ce délai est de 8,2 jours avec des extrêmes allant d'une survenue immédiate en sortie de CEC à 65 jours du postopératoires. Concernant la cardiopathie congénitale, le délai moyen de survenue du BAV est de 183,25 jours avec des extrêmes allant d'une survenue immédiate en sortie de CEC à 1245 jours (3 ans et 5 mois). Le BAV est survenue à J1 du post opératoire chez le patient ayant eu un pontage aorto coronaire. 2 patients ont présenté un BAV complet régressif; âgés respectivement de 45 ans et de 43 ans ayant eu un remplacement valvulaire mitral et d'un double remplacement mitro-aortique à J1 et J2 du post opératoire. Une seule patiente opérée pour un canal atrioventriculaire (CAV) a présenté en post opératoire un BAV premier degré immédiat persistant dégénérant en BAV complet après 2 ans de l'intervention chirurgicale.

**Figure 4:**
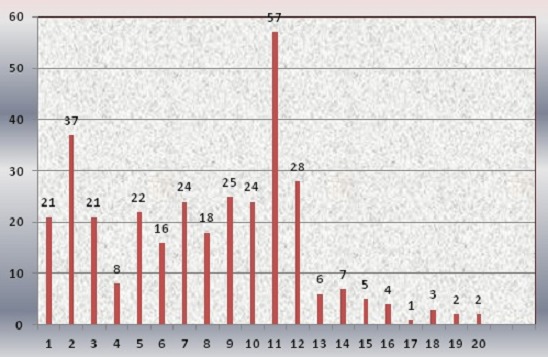
Répartition des malades selon le délai d'implantation du pacemaker (Jours)

Sur les 23 malades colligés, 20 patients ont eu une implantation définitive d'un pacemaker. Le délai d'implantation par rapport à la date de la découverte du BAV varie dans notre série d'une implantation immédiate après le diagnostic positif (J0) à un délai d'implantation allant jusqu’à 57 jours avec un délai moyen de: 16 j (Figure 4). Les suites opératoires après implantation du pacemaker étaient simples chez 19 des 20 patients implantés. Un hématome de la loge musculaire repris chirurgicalement a compliqué l'implantation d'un pacemaker chez un patient âgé de 44 ans ayant eu un double remplacement mitro aortique avec plastie tricuspidienne sous traitement anti coagulant.

## Discussion

Le bloc auriculo-ventriculaire a été rapporté comme étant la brady-arrhythmie la plus commune nécessitant la mise en place d'un pacemaker après chirurgie cardiaque. Sa survenue est imprévisible et son incidence varie selon la présence ou non de facteurs de risque et selon le type d'intervention chirurgicale. L'incidence du BAV compliquant la chirurgie des cardiopathies congénitales est de 1-3% malgré les progrès chirurgicaux et malgré l'expérience des chirurgiens. Le risque de développer des troubles conductifs après chirurgie valvulaire est variable selon les séries; il est rapporté entre 23 et 37% [1, 2]. La détermination du délai exact du retour à la normale de la conduction auriculo-ventriculaire n'est pas encore possible et cette résolution est souvent imprévisible [2]. Ainsi le BAV post opératoire est souvent transitoire avec une résolution complète avant le dixième jour [3]. Cette résolution se fait vers le 9ème jour après chirurgie des cardiopathies congénitales [1]; elle est plus précoce après chirurgie valvulaire (environ une semaine). Dans notre série 2 patients ont présenté un BAV complet régressif, âgés respectivement de 45 ans et de 43 ans, ayant bénéficié d'un remplacement valvulaire mitral et d'un double remplacement mitro-aortique à J1 et J2 du post opératoire soit: 8,7% par rapport à l'ensemble de la cohorte et 43,7% de l'ensemble des malades valvulaires.

La régression du trouble conductif au delà de la première semaine n'est pas toujours synonyme de guérison, et elle ne peut être qu'un changement du mode d'expression du BAV qui devient alors paroxystique, constituant dans ce cas un marqueur important de survenue de BAV tardif. Plusieurs facteurs surtout la présence de troubles résiduels du système de conduction jouent un rôle important dans la détermination du risque tardif de mortalité et morbidité chez les patients ayant eu un BAV transitoire [4]. Deux des trois patients chez qui le bloc de branche a été diagnostiqué ont développé un BAV complet à quelques années du post opératoire. D'après certains auteurs l’étude endocavitaire est réalisée systématiquement chez tout patient ayant présenté un BAV II Mobitz II, un bloc tri fasciculaire ou ayant eu un bloc complet durant plus de 48 heures du post opératoire et gardant des anomalies de la conduction [5]. Le BAV de haut degré peut être d'apparition tardive sans expression en post opératoire immédiat, son incidence a été décrite dans la littérature. Lin et al. ont rapporté une incidence entre 0.3 et 0.7% après chirurgie des cardiopathies congénitales avec un délai moyen d'apparition à 4,1 ans [6]. C'est une complication rare mais sérieuse survenant après réparation chirurgicale d'une CIV avec un délai d'apparition de 4 ans du post opératoire [7]. Dans notre série, 12 patients ont présenté un BAV tardif, soit 52,2% des malades avec un délai moyen d'apparition de 189 jours et des extrêmes allant de 3 à 1245 jours (3 ans et 5 mois). Cette notion de survenue tardive de BAV post chirurgie cardiaque doit être prise en considération concernant le suivi des patients qui sont dans ce cas à haut risque de mort subite.

La résolution tardive du BAV post chirurgie cardiaque a été décrite dans plusieurs séries. Elle est définie par la disparition du trouble conductif au 14ème jour du post-opératoire [2]. Concernant notre série 17% de l'ensemble des malades ont présenté une résolution tardive du BAV, cette incidence était de l'ordre de 9% chez les patients suivis pour cardiopathies congénitales. Plusieurs facteurs de risques déterminent la survenue du BAV post chirurgie cardiaque. On distingue des facteurs de risque liés à l'atteinte anatomique per opératoire du système de conduction notamment du noeud auriculo-ventriculaire qui est dans ce cas responsable de l'apparition de troubles conductifs entre autres d'un BAV. Elle est expliquée essentiellement par deux mécanismes:La proximité du site de l'intervention chirurgical par rapport au noeud auriculo-ventriculaire induisant une atteinte du système conductif et l'ischémie myocardique responsable d'une ischémie du système de conduction. Une corrélation significative a été démontrée entre la survenue de troubles conductifs, notamment le BAV et le type des cardiopathies congénitales opérées, ceci est vrai pour trois types d'interventions: communication inter ventriculaire, tétralogie de Fallot, canal atrio-ventriculaire [2]. La complexité de la procédure chirurgicale est un facteur prédictif important de survenue de ce trouble conductif. D'autres cardiopathies congénitales sont également pourvoyeuses de troubles conductifs à savoir les corrections chirurgicales d'une transposition de gros vaisseaux ou celles palliatives de l'atrésie tricuspide ou l'intervention de Fontan qui consiste en une anastomose atrio-pulmonaire [8]. Dans notre série Les cardiopathies congénitales représentent 52% de l'ensemble des indications opératoires. 50% des patients sont opérés pour une communication inter-ventriculaire. Le canal atrio-ventriculaire représente 33,3% de l'ensemble des indications, suivi de la transposition des gros vaisseaux avec 16,6%, la sténose pulmonaire ainsi que la sténose sous aortique ne représentent respectivement que 4,3% chacune. Il a été rapporté que les patients atteints de trisomie 21 opérés pour une cardiopathie congénitale présentent plus de risque de survenue de troubles conductifs en post opératoire que les patients opérées pour les mêmes cardiopathies non atteints de trisomie 21. Ainsi La relation anatomique entre les anomalies de conduction au cours de la trisomie 21 et le système de conduction au cours de la communication interventriculaire (CIV) péri membraneuse a été décrite mais aucun facteur favorisant n'a encore été retenu dans la littérature [8]. Tucker et al. conclu dans la même série que le fait que les patients trisomiques opérés pour une CIV péri membraneuse sont d’âge plus jeune et de poids plus faible par rapport aux patients opérés pour la même pathologie ne présentant pas de trisomie 21, n'explique pas l'incidence élevée de BAV au cours de cette série [8]. Selon Tucker, ceci suggère qu'il existe un autre facteur lié à la trisomie 21 expliquant cette incidence. Dans la littérature aucune étude n'a encore été faite dans ce sens.

Concernant la chirurgie valvulaire le risque de survenue de BAV post opératoire est plus important. Ce risque augmente avec le remplacement valvulaire, la chirurgie multi valvulaire. Plusieurs études ont démontré la fréquence élevée des BAV nécessitant une stimulation définitive après chirurgie aortique qu'après chirurgie mitrale [9]. Dans notre série la cardiopathie valvulaire représente 43,7% de l'ensemble des indications opératoires. 80% des patients valvulaires sont opérés pour un remplacement valvulaire aortique. Ce risque peut être expliqué par la proximité du site d'intervention chirurgicale avec le système de conduction atrio-ventriculaire et peut être majoré par la présence de calcifications annulaires aortiques, d'abcès de l'anneau aortique, de rétrécissement aortique calcifié ou de bicuspidie. Plusieurs études ont montré que la chirurgie valvulaire tricuspidienne isolée ou associée à une chirurgie poly valvulaire augmente de manière significative le risque de survenue de BAV. Dans notre série, la plastie tricuspidienne a été réalisée chez la moitié des valvulaires. L'ischémie myocardique per opératoire prolongée est reconnue comme responsable de la survenue de BAV post opératoire de même qu'une mauvaise protection myocardique. Le mécanisme exacte n'est pas encore clairement établi. Il a été rapporté que les propriétés intrinsèques du système de conduction du noeud auriculo-ventriculaire procurent une protection moindre dans les situations entrainant une ischémie [10]. Une augmentation du temps du clampage aortique ainsi que de la durée de CEC sont susceptible d′augmenter le risque de survenue de BAV post chirurgie cardiaque essentiellement au cours des cardiopathies congénitales.

Comme autres facteurs de survenue d′ischémie myocardique, l'utilisation pré opératoire du sotalol ou d′amiodarone peut augmenter de manière significative l'incidence du BAV post chirurgie cardiaque. Ces deux drogues prolongent le temps du clampage aortique. Elles possèdent des propriétés pro- arythmiques et leur utilisation peut influencer le fonctionnement normal du système de conduction [11]. L'utilisation préopératoire de bétabloquant et de la digoxine a été décrite comme étant responsable d'une augmentation du taux de troubles conductifs après chirurgie cardiaque. Les patients ayant été mis sous digitalique initialement pour améliorer la fonction ventriculaire, ont par conséquent une augmentation du volume ventriculaire exerçant ainsi une tension sur le système conductif. D'autres séries n'ont pas, par contre, retenu ces deux facteurs comme responsable de l'augmentation de l'incidence de troubles conductifs. Aucun de nos malades n'a été mis sous digitalique en pré opératoire. L'hypothermie systémique per opératoire est souvent liée à l′ischémie myocardique. Elle est d′ailleurs plus prononcée chez les patients présentant un BAV post opératoire. C'est un facteur d'atteinte directe du noeud auriculo-ventriculaire: la profondeur de l'hypothermie a été associée à plus de dommages au niveau du tissu de conduction, à une diminution de la protection myocardique et de l'activité électrique [2]. L'association entre une déviation axiale gauche préopératoire et la survenue de troubles conductifs nécessitant une stimulation cardiaque définitive a été récemment décrite [12]. En effet, les patients ayant développé des troubles conductifs ont une incidence d′une déviation axiale gauche plus importante par rapport aux autres malades. D'autres facteurs de risque ont été étudiés. Ainsi la présence en pré opératoire d'un bloc de branche droit ou gauche est un facteur prédictif indépendant et important du risque de mise en place d'un pacemaker suite à l′apparition de troubles conductifs [2]. Dans notre série, 6 patients avaient un bloc de branche incomplet, soit: 26% de l'ensemble des malades, dont 2 patients présentant un bloc de branche droit. Dans certaines études, les patients ayant présenté des troubles conductifs notamment un BAV post chirurgie des cardiopathies congénitales sont d’âge jeune et de poids faible [13].

Concernant le délai d'implantation du pacemaker le moment idéal constitue jusqu’à présent un sujet de controverse. Dans la série de Ben Ameur et al, les implantations de pace maker définitifs ont été pratiquées en moyenne 31,8 jours après l'intervention (8-128 jours) [14]. Classiquement, ce délai est de deux à trois semaines par rapport à l'intervention chirurgicale, mais il reste toujours discuté. Il est légitime de considérer qu'en cas de facteurs importants, particulièrement, lorsqu'il s'agit d'une chirurgie aortique ayant de fortes chances d'avoir endommagé le faisceau de His et lorsque les troubles apparaissent d'emblée et durent au delà de 48 h, que les délais peuvent être raccourcis. Donc un appareillage est préconisé vers la fin de la première semaine. La persistance ou non du BAV post opératoire est un facteur déterminant pour la mise en place du pacemaker. Le BAV post opératoire 2ème et 3ème degré non résolu ou persistant au delà de 7 jours est une classe 1 d'indication du pacemaker et Il n y a pas d'indication à la mise en place du pacemaker chez les patients ayant eu un retour à la normal de la conduction A-V [15]. Certains auteurs soutiennent l'idée d'une implantation plus précoce. Hancock et al ont proposé que la présence d'un BAV complet durant 3 jours dans le post opératoire est une indication raisonnable pour la mise en place du pacemaker [16]. Kim et al proposent dans une série portant sur 155 patients opérés pour valvulopathie que le délai d'implantation définitif d'un pacemaker chez les patients présentant un BAV complet dans les 24 heures post intervention et persistant plus de 48 heures ne doit pas excéder sept jours [17]. D'autres auteurs soutiennent la même suggestion. L'indication de la mise en place du pacemaker au cours d'un BAV transitoire reste toujours controversée. Elle est recommandée chez les patients avec BAV transitoire ayant gardé des troubles conductifs résiduels.

À la lumière de ces données, certains auteurs suggèrent un appareillage précoce vers la fin de la première semaine postopératoire lorsque le trouble conductif persiste et s'associe à des facteurs prédictifs. Le délai d'implantation par rapport à la date de la découverte du BAV variait dans notre série d'une implantation immédiate après le diagnostic positif (J0) à un délai d'implantation allant jusqu’à 57 jours avec un délai moyen de: 16 j. Concernant les cardiopathies valvulaires, le délai moyen d'implantation a été de 20,2 jours avec des extrêmes de 8 à 28 jours. Le délai moyen d'implantation au cours des cardiopathies congénitales était de 13,7 jours avec des extrêmes de 1 à 57 jours.

## Conclusion

La chirurgie cardiaque sous circulation extra corporelle conduit à un risque non négligeable de troubles conductifs auriculo-ventriculaires. Ils représentent des séquelles lourdes et graves compromettant le pronostic de la maladie et conduisant parfois à l'implantation d'un stimulateur cardiaque définitif. Le mécanisme de ce trouble conductif reste mal élucidé, témoignant des difficultés de sa prise en charge, et plus particulièrement du moment exact de l'implantation du pacemaker. De ce fait, il est primordial d'identifier d'une part les facteurs prédictifs et d'autre part de comprendre leur mécanisme. Plusieurs facteurs ont été rapportés dans la littérature, liés essentiellement à l'atteinte anatomique du système de conduction ou par l'atteinte directe de l'artère du noeud A-V du réseau coronaire ou par un temps du clampage aortique de CEC allongé ou d'hypothermie profonde. Classiquement les délais d'implantation du stimulateur cardiaque définitif par rapport à l'intervention chirurgicale sont de deux à trois semaines mais ces délais restent controversés par certains auteurs qui prônent des implantations plus précoces dans certaines situations à risque.
